# Being pro-active in meeting the needs of suicide-bereaved survivors: results from a systematic audit in Montréal

**DOI:** 10.1186/s12889-020-09636-y

**Published:** 2020-10-10

**Authors:** Fabienne Ligier, Jessica Rassy, Gabrielle Fortin, Ian van Haaster, Claude Doyon, Charlie Brouillard, Monique Séguin, Alain Lesage

**Affiliations:** 1 Centre Psychothérapique de Nancy, Pôle Universitaire de Psychiatrie de l’Enfant et de l’Adolescent, 1 rue du Dr Archambault, F-54520 Laxou, France; 2 EA 4360 APEMAC, Université de Lorraine, Vandoeuvre-lès-Nancy, France; 3 School of Nursing, Université de Sherbrooke, Longueuil, Canada; 4 Centre de recherche de l’Institut Universitaire en Santé Mentale de Montréal, Montreal, Canada; 5 CIUSSS de l’Est de l’Ile de Montréal, CLSC St-Michel, Montreal, Canada; 6 Department of Psychoeducation and Psychology, Université du Québec en Outaouais, Gatineau, Canada; 7 Centre intégré de santé et service social de l’Outaouais (CISSSO), Outaouais, Quebec, Canada; 8 Department of Psychiatry, Université de Montréal, Quebec, Canada

**Keywords:** Bereaved, Help seeking, Needs, Postvention, Suicide

## Abstract

**Background:**

Suicide is a major public health concern. In 2017, the suicide rate in Canada was 11 per 100,000 inhabitants. According to literature, 1 in 5 people have experienced a death by suicide during their lifetime. The aim of this study was to describe the met and unmet needs of suicide-bereaved survivors and to provide postvention recommendations.

**Methods:**

Further to an exploratory mixed-method audit of 39 suicides that occurred in Montreal (Canada) in 2016, suicide-bereaved survivors (*n* = 29) participated in semi-structured interviews and completed instruments to discuss and assess potential pathological grief, depression (PHQ-9), and anxiety (GAD-7), as well as health and social services utilization. A panel then reviewed each case and provided recommendations. The mean age of participants was 57.7 years and 23 were women.

**Results:**

Although help was offered initially, in most cases by a health professional or service provider (16/29), 22 survivors would have liked to be contacted by telephone in the first 2 months post suicide. Four categories of individual unmet needs (medical/pharmacological, information, support, and outreach) and one collective unmet need (suicide pre/postvention training and delivery) emerged.

**Conclusions:**

Although Quebec provincial services have been developed and offered to suicide-bereaved survivors in the past decade, many dwindled over time and none has been applied systematically. Recommendations for different stakeholders (Ministry of Health and Social Services, coroners, NGOs, and representatives of suicide-bereaved survivors) outlined in this study could be an interesting first step to help develop a suicide pre/postvention strategy.

## Background

Suicide is a major public health concern. In 2017, the suicide rate in Canada was 11 per 100,000 inhabitants [1]. According to literature, 1 in 5 people have experienced a death by suicide during their lifetime [2–4].

The grief experienced by suicide-bereaved survivors (SBS) is very specific. Dealing with a loved one’s suicide can be extremely challenging for SBS, to the point that they may become more vulnerable to an array of problems [5]. The majority of SBS will not develop pathological bereavement or other problems. However, it is important to recognize those who do and to bear in mind that this may occur even 18 to 24 months post suicide [6]. Bailley et al. [7] found that, compared with bereaved individuals in other contexts, SBS were more likely to experience feelings of rejection and guilt, suggesting that they also felt higher levels of shame and stigma. This context of death can have an impact on the bereavement process, the development of somatic or mental health disorders and, ultimately, suicide risk [8–11]. McMenamy and al [12]. found that SBS had a high level of psychological distress, guilt, anxiety, depression and trauma signs.

Studies have found that SBS needs for help were met in different ways. SBS received help from friends and family, general or suicide grief support groups, individual psychotherapy, information and referral services, talking one to one with another suicide survivor, and psychotropic medication [12–14].

Bereavement programs do exist and some have been assessed. However, there is little knowledge relating to the content and effectiveness of these programs [15]. Some evidence of benefits has emerged from a few intervention studies [15–17]. For example, Andriessen et al. [2] carried out a systematic review of controlled studies to assess evidence of the effectiveness of interventions for SBS and simultaneously appraise research quality. Their results showed that interventions targeting support, therapy and education seemed to work best when they included the social environment of the bereaved and when the therapy sessions were led by professionals. In another systematic review, Linde et al. [16] found that bereavement groups had positive effects on uncomplicated grief and that cognitive-behavioral programs had positive effects on individuals at risk for suicide. However, as pointed out by Andriessen et al. [2] and Linde et al. [16], the overall quality of research in the field of SBS postvention remains very weak. That being said, the current available evidence on the effectiveness of these interventions is neither strong nor reliable. For example, the results of a systematic review of the literature by Szumilas and Kutcher showed that only 16 of 49 studies of suicide postvention programs met their inclusion criteria regarding quality and evidence of effectiveness [17]. Outcomes and measures varied widely from one study to another. Given the absence of any evidence-based suicide postvention program, further research is required into the exact form and structure of these programs. Furthermore, if these programs are proved ineffective, it could be because they were not based on the actual needs of bereaved adults to begin with. As shown by Wilson and Marshall [18], less than half of individuals bereaved by suicide who expressed needing help with their grief process actually received help and only 40% of these were satisfied with the help received. In a study exploring the needs of SBS in Ontario (Canada), Gall, Henneberry, and Eyre [19] described and compared the perspectives of SBS and of mental health workers and found that they complemented each other and helped identify best practices for SBS postvention. Pitman et al. [20], too, demonstrated the importance of addressing support needs from the perspective of SBS. Finally, Castelli-Dransart and Séguin [21] recommended that the help offered to SBS follows a specific progression according to needs: family support in the first weeks, support group if necessary, and therapy for difficult or pathological bereavement.

Finally, SBS often have difficulty seeking help because of depression or lack of energy. They may also have lost confidence in the health and social services systems that failed their loved one or because they feel shame over what happened [7, 9, 12, 22–24]. SBS have also underlined in some studies that there was a lack of information about where to find resources and that resources were not always available [13].

The above studies and findings argue in favor of approaching SBS needs proactively [25–27] and of developing specific programs to address these needs. Moreover, it is reassuring to know that programs exist that have proved effective in the very specific situations of complicated bereavement [28, 29].

In Canada, health and social services are provincial responsibilities. In the province of Quebec, suicide prevention centers often offer help in the form of support groups to the community. But it seems that SBS are not systematically referred to these centers. This is why it is important to assess social and health service needs and to understand why such needs go unmet. Grieving difficulties, service utilization, and unmet needs should be assessed systematically.

Against this background, we undertook an exploratory cross-sectional and retrospective mixed-method study to describe the met and unmet needs of SBS 2 years after the event and to formulate specific suicide postvention recommendations over this two-year period.

## Methods

This study consisted of a secondary analysis of data collected from SBS outreached in the course of a systematic audit of the consecutive suicide cases that occurred in the east end of the island of Montreal in the first 10 months of 2016 [30]. The method of this audit was based on the method used in an audit conducted in 2002–2003 in New Brunswick, Canada [13]. The research protocol was approved by the Scientific Committee of the Montreal Mental Health Institute (November 6, 2018) and the publication of results was approved by its Ethics Research Committee (February 1, 2019), project number 2019–1647.

### Sample and inclusion procedure

The sample consisted of individuals bereaved by suicides that occurred in 2016. Sixty individuals died from suicide in 2016 in the east end of the island of Montreal (population rate of 11.2 per 100,000). Of these, 39 consecutive cases were audited, corresponding to the cases in the first 10 months of 2016. For financial reasons, we had to terminate the audit before having examined all the cases that occurred in 2016. Individuals who died by suicide in 2016 had been securely identified by the Quebec Coroner’s Office (QCO). The SBS noted as contact resources in the file of these individuals (e.g., family member, friend, and people living in the same household) were initially contacted by the QCO by mail, between the end of 2017 and September 2018, that is, about 2 years after the loss. There were no inclusion or exclusion criteria. The letter that they received explained the purpose of the audit and solicited parties interested in participating in it. Participation was voluntary, no incentives were offered, and no obligations were imposed. Interested parties were then contacted to schedule an interview. Interviews were conducted at their home, at the research center or over the telephone, according to the participant’s preference and availability. For the purposes of this study, we used only the second part of the interview that focused specifically on the SBS’s experience of the suicide rather than on the suicide victim. Twenty-nine survivors bereaved by 27 individuals who died by suicide in the east end of the island of Montreal in 2016 agreed to be interviewed in 2018–2019. In two cases, we interviewed two members of the family (a parent and a brother in one case, a brother and a sister in the other). Mean age of participants was 57.7 years (*SD* = 13.1). Of the 29, 23 were women. Sample characteristics are given in Table 1.

**Table 1:** Initial Characteristics of Suicide-Bereaved Survivors (*N* = 29)

Characteristics	Suicide-bereaved survivors
	*N* = 29
	Raw number (percentage)
Employed, yes	14 (48.3)
Married or in a common law relationship	11 (37.9)
Children, yes	28 (96.6)
Relationship to suicide victim	
**•** Parent	7 (24.1)
**•** Child	7 (24.1)
**•** Sister or brother	7 (24.1)
**•** Spouse or ex-spouse	6 (20.7)
**•** Other (friend or nephew)	2 (6.9)
Family history of completed or attempted suicide	
**•** Completed suicide, yes	8 (27.6)
**•** Attempted suicide, yes	4 (13.8)
**•** Own history of attempted suicide, yes	2 (6.9)

### Data collection

Data were collected through semi-structured interviews and two self-report instruments (See supplementary files 1 and 2). Aside from socio-demographics, data were also collected on risk/protective factors of pathological grief, such as relational proximity to the deceased. SBS were asked open-ended questions regarding their emotional experiences post suicide death notification and how long they lasted. General physical health, mental health and problems/difficulties post suicide were also noted. We also asked an open-ended question to investigate presence of personal or family history of suicide, which has been identified as a suicidal risk factor. We constructed a semi-structured interview guide from the five items of the Brief Grief Questionnaire, which we translated into French but have yet to validate [31], and other items based on the literature. We also asked participants about the usefulness of resources that might have used, such as their general practitioner. Depression and anxiety were assessed through self-reported data on the Patient Health Questionnaire (PHQ-9 [32–34];) and the Generalized Anxiety Disorder (GAD-7 [35, 36];), respectively. During the interview, the audit team member explored whether other SBS might be interested in participating in the study, as more than one SBS could be interviewed regarding the same case. Names provided were contacted and invited to be interviewed.

The unmet needs assessment method was developed by the MRC Social Psychiatry Unit of the Institute of Psychiatry [37]. Under this methodology, if an individual is socially disabled in association with a mental disorder for which an effective and acceptable form or model of intervention exists, be it for corrective or preventive purposes, then that individual is in need of that intervention. There is an unmet need when such intervention is not provided. Unmet needs were identified by the SBS themselves during the interview as well as by the audit panel in the course of its review of the case. This panel was composed of 10 individuals, including clinicians (physicians, psychiatrists, psychologists and nurses), health and social service managers, a provincial representative of a non-governmental suicide prevention organization (NGO), and a retired judge who represented SBS. A vignette was drafted based on the information provided during the interview by each SBS. The panel reviewed the vignette (about 1 to 2 h per vignette) over the course of a monthly meeting and provided recommendations.

### Data analysis

JR used content analysis [38] to identify unmet needs and group them in a small number of categories. Content analysis was a three-stage process: 1) the immersion stage allowed the researcher to get acquainted with the data more in depth; 2) the reduction stage served to simplify the data and extract themes; and 3) the interpretation stage served to draw conclusions on the final findings [38]. FL and AL confirmed the findings. Descriptive statistics compiled included raw numbers, percentages, means and standard deviations. Analyses were carried out on SAS 9.4 software.

## Results

### Quantitative results

The average rating of SBS for relational proximity to the individual who died by suicide on a scale of 0 (no relationship) to 10 (highest relational proximity) was 8 (*SD* = 1.9).

In most cases (51.7%), the SBS were informed of the suicide by police officers. Upon doing so, some officers offered help verbally or handed over a suicide prevention center information card, at their discretion. Of the ten SBS who went to general practitioners for help, eight found it useful and four were prescribed medication. Further details regarding death notification, offers of psychotherapy/counselling, and utilization of professional, community organization and social network support and services are presented in Table 2.

**Table 2:** Death Notification, Initial Offer of Help, Support and Services Received (*N* = 29)

Characteristics	Suicide-bereaved survivors
	*N* = 29
	Raw number (percentage)
Source of suicide death notification	
**•** Police	15 (51.7)
**•** Found suicide victim	9 (31)
**•** Family member	2 (6.9)
**•** Other	3 (10.3)
Offer of help	
**•** Initial help offered by professionals	16 (55.2)
○Police	4 (13.8)
○Funeral home	2 (6.9)
○General practitioner	1 (3.4)
○Social worker	2 (6.9)
○Associations	2 (6.9)
○Other individuals	5 (17.2)
**•** No initial offer of help	13 (44.8)
Type of psychological counselling proposed	
**•** Group for SBS	5 (17.2)
**•** Group for bereaved individuals in general	1 (3.4)
**•** Individual psychotherapy by psychologists	3 (10.3)
**•** No specific psychological intervention proposed	20 (69.0)
Type of professional, community and social network support reported (more than one source possible)	
**•** Healthcare professional	19 (65.5)
**•** Non-profit suicide prevention organization	8 (27.6)
**•** Other	2 (6.9)
**•** Family support	21 (72.4)
**•** Support from friends	18 (62.1)
**•** Support from neighbors	10 (34.5)
**•** Support from colleagues	6 (20.7)
**•** No support reported	2 (6.9)
**•** No information	1 (3.4)

The vast majority of the SBS did not screen positively for depression or anxiety at time of interview on the PHQ-9 and the GAD-7. The mean scores (*SD*) on the instruments were 4.56 (4.08) and 2.63 (3.54), respectively. A score of 10 or more is indicative of high risk for major depression and anxiety disorder. Table 3 presents the various emotional experiences reported by the SBS in the 2 years post suicide, which included shock, sadness and anger. In addition, of the SBS who witnessed the suicide (*n* = 2) or found the deceased (*n* = 9), three reported insomnia and six had flashbacks. Moreover, 22 SBS did not feel rejected by the loved one who died by suicide and 17 still wondered why their loved one committed suicide but without the questioning being obsessive in any way. Some SBS also reported developing a somatic condition (*n* = 8/29) or a psychiatric disorder such as anxiety or depression (*n* = 3/29). Furthermore, 3/29 increased their alcohol consumption and 5/29 increased their tobacco consumption post suicide. One of these last five also developed depression.

**Table 3:** Emotional Experiences of Suicide-Bereaved Survivors and Duration in Two Years Post Suicide (*N* = 29)

Emotional experiences	No. of SBS/29 (%),Mean duration (*SD*)
Emotional experiences post suicide death notification	
**•** First = “shocked”	21 (72.4), 20 days (31)
**•** Second = “sad/discouraged”	12 (41.3), 219 days (35)
**•** Third = “angry”	4 (13.7), 83 days (62)
Present feelings	
**•** “At peace”	9 (31.0%)
**•** “Sad, anxious”	5 (17.2%)
**•** “Loss”	3 (10.3%)

### Qualitative results

Six SBS who did not receive professional help post suicide felt they might have benefitted from such help. In all, 22 SBS would have liked to receive a call from a professional within 66 days (*SD* = 65) on average of the suicide. Table 4 summarizes the main needs to emerge from the content analysis of the SBS interview data, indicates who should meet these needs and presents the recommendations provided by the audit panel for each need. We analyzed the needs and the recommendations in order to project the individual clinical and social needs of the SBS against the existing resources of the provincial health and social services system and existing regional and local programs using the quality of care matrix model [39]. Some of these needs were considered met by the SBS, others were not. However, either way, the SBS and/or the audit panel deemed that they should inform future postvention for SBS.

**Table 4:** Needs of Suicide-Bereaved Survivors, Who Should Meet Needs, and Recommendations (With Number of Cases Supporting Recommendation)

Need	Who should meet need	Recommendation
1. Medical care and pharmacological needs (*n* = 5)	Quebec Ministry of Health and Social Services, Quebec College of Physicians, Quebec Order of Nurses	Facilitate SBS access to medical and pharmacological care (access to physicians and/or nurse practitioners).
2. Initial need for information (*n* = 2)	First responders	Police forces and other first responders should inform SBS of how and where to access support resources.
3. Support needs (*n* = 17)	Quebec Ministry of Health and Social Services, regional health and social services agencies, Quebec College of Physicians, Quebec Order of Nurses, Quebec Order of Psychologists, Quebec Association for Suicide Prevention, Quebec Ministry of Public Security	Refer SBS to individual, family and/or support groups.
		Develop a protocol for systematic identification, care and follow-up of SBS.
4. Outreach needs (*n* = 15)	Quebec Coroner’s Office, Quebec Ministry of Health and Social Services, Quebec Association for Suicide Prevention, Quebec Ministry of Public Security	Answer questions and provide information about available resources at time of suicide death notification.
		Reach out to SBS in first 6 months post suicide and offer a follow-up to see how they are doing, provide proper documentation and refer them to available resources or qualified mental health professionals according to their needs.
		Pay close attention to financial and psychosocial needs, especially from a cultural perspective, and refer to proper resources.
5. Needs for suicide pre/postvention training and delivery (*n* = 4)	Quebec Ministry of Health and Social Services, regional health and social services agencies, Quebec College of Physicians, Quebec Order of Nurses, Quebec Order of Psychologists, Quebec Association for Suicide Prevention, first responders	Organize suicide prevention training for frontline and specialist health and social services professionals.
		Raise awareness of how postvention with SBS can prevent suicide.
		Offer postvention to healthcare teams.

## Discussion

Our findings show that many SBS needs were not met or failed to be addressed by existing bereavement programs. In the majority of cases, the SBS learned of the suicide from police services. However, one third of the SBS were the ones who found the deceased. Offers of help came from different sources, including police officers, funeral homes, and general practitioners. However, nearly half of the SBS were offered no help. The multiplicity and diversity of offers indicate that there is no systematic program for SBS and that the help offered is at the discretion of the person offering the help. Under the circumstances, SBS are likely to be treated differently and unevenly. The majority of SBS found support in their social network and even if they went through a hard time initially, they were not afflicted by pathological bereavement, depression or anxiety 2 years post suicide. However, some SBS would have liked to be contacted by telephone by a professional and six SBS who received no help post suicide felt that they might have benefited from receiving such help. This underscores the importance of being proactive with this vulnerable population from time of suicide death notification to at least 1 year post suicide. A proactive approach is often needed to offer help to these vulnerable individuals [25]. Also, such offers must be repeated at intervals because individuals experience grief differently and may need support at different times [25, 26]. According to McKinnon and Chonody [27], proactive help should be offered by first responders, coroners and other professionals that come into contact with SBS very early on. The absence of a formal process to connect SBS with support programs is in fact the principal unmet need of SBS [27]. The needs and recommendations that emerged from this study underscore just how important it is for SBS programs to be interprofessional and to involve a broad array of stakeholders, including the QCO, law enforcement services, NGOs and the provincial ministry of health and social services.

Though results shed fresh light on the experience of SBS, our study is not without limitations. First, this was a retrospective study prone to memory and reconstruction biases. However, most of the SBS interviewed were able to describe their emotional experiences 2 years post suicide and, as is often the case, ordeals tend to render more vivid memories. Second, although all interviews were recorded, they were not transcribed. SBS needs were garnered from vignettes constructed by the research team for each case. Third, the study focused only on SBS mentioned in police reports; other family members, friends and colleagues were neither identified nor contacted. However, the research team sought to contact other relatives of the deceased as well by asking direct family members whether anyone among family and friends might want to participate in the study. Finally, the multidisciplinary panel’s recommendations regarding unmet needs remain in the end hypotheses to be put to the test of a new program implementation [37].

Our study also has its strengths. First, the recommendations were formulated by an audit panel of health professionals and SBS, as recommended by Gall et al. [19]. To our knowledge, SBS needs have never been assessed to this extent, that is, by interviewing SBS and gathering the views and recommendations of an audit panel. Second, we were able to triangulate the data thanks to the various sources of data used. This enhanced the credibility, transferability and dependability of the study results [40]. Third, all the SBS were interviewed systematically, including those who did not seek help. This is particularly interesting given that some SBS who did not seek help were able to identify unmet needs and make recommendations for bereavement programs.

The results of our study are consistent with the literature. Indeed, as underlined by Pitman et al. [20] and McKinnon and Chonody [27], programs need to be proactive. SBS would like to receive a call because it is sometimes very difficult to seek help. Moreover, help should be offered at different times given that some individuals would prefer being contacted by telephone in the days following the suicide death notification, others after 4 months, and others still after 6 months. Pitman et al. [20] reported similar results.

## Conclusion

Although services have been developed and offered at the provincial and regional levels to suicide-bereaved survivors in the past decade, many dwindled over time and none has been applied systematically, suggesting that the 15-year old Quebec suicide prevention plan is perhaps ripe for review [41]. These services include having police officers give SBS suicide prevention center information cards at time of suicide death notification, having suicide prevention centers systematically call SBS after receiving contact information from the QCO, and offering bereavement services and suicide prevention and postvention training to professionals in schools. To ensure the survival of these strategies and offer better help and support, a provincial program to systematically monitor SBS is necessary. This program would need to be proactive and monitor SBS for 2 years post suicide. Ideally, it should be developed collaboratively by the Quebec Ministry of Health and Social Services, a suicide prevention NGO and the QCO, and should be financed jointly by the Quebec Ministry of Health and Social Services and the Quebec Ministry of Justice. It could then be monitored and evaluated by a coalition for suicide prevention that would include SBS representatives and researchers. These recommendations are an interesting first step to help the Quebec Ministry of Health and Social Services develop and implement a systematic suicide pre/postvention strategy.

## Supplementary information

**Additional file 1: Supplementary file 1.** English Version of the semi-structured interview, GAD-7 and PHQ-9.

**Additional file 2: Supplementary file 2.** French Version of the semi-structured interview, GAD-7 and PHQ-9.

## Data Availability

The datasets generated and analyzed during the current study are available from the corresponding author on reasonable request.

## Abbreviations

SBS: Suicide-Bereaved Survivors
QCO: Quebec Coroner’s Office
