# Supplementary material for: Being pro-active in meeting the needs of suicide-bereaved survivors: results from a systematic audit in Montréal
Source: BMC Public Health. 2020 Oct 10;20:1534. doi: 10.1186/s12889-020-09636-y (PMC7547412; doi:10.1186/s12889-020-09636-y)
Supplement: Supplementary file 1 — Additional file 1: Supplementary file 1. English Version of the semi-structured interview, GAD-7 and PHQ-9. [file 12889_2020_9636_MOESM1_ESM.docx]

**SYSTEMATIC AUDIT OF SUICIDE CASES FROM THE CIUSSS OF THE “L’EST-DE-L’ILE DE MONTREAL”**

**Questions for the suicide-bereaved survivor**

(Questions to ask after having talked about the person who died by suicide)

Age and gender of the person who died by suicide: ……………………………………………

Link with the suicide-bereaved survivor: ……………………………………………………

*« Now, we are going to talk about you, about what you have faced these last months, in order to try to understand how we could organize services to meet the needs of persons who are in the same situation than you”*

**Age: …………………………..**

**Gender:** Male Female

00

00

**1/ Are you working?**

Yes No

00

00

**2/ What is your marital status?**

In relationship Divorced or separated Widow(er)

Single

**3/ Do you have children?**

Yes No

00

00

If Yes, how old is he/she - are they?

……………………….

……………………….

……………………….

……………………….

How often do you meet them?

………………………………

………………………………

**4/ (About the suicide)**

Do you have other relatives than ….……… who died by suicide ?

Yes No

00

00

If Yes, who (degree of kindship) and at what age?

……………………….

……………………….

Do you have other relatives who made a suicide attempt?

Yes No

00

00

If Yes, who (degree of kindship) and at what age?

……………………….

……………………….

Did you make a suicide attempt?

Yes No

00

00

If Yes, at what age? (*Note every age if several suicide attempts*)

……………………….

……………………….

**5/ On a 0 to 10 scale, how would you assess your relational proximity with ……? (note the number given)**

00

00111&&

**6 et 7/ GAD-7 and PHQ-9**

Available on

[https://www.torbayandsouthdevon.nhs.uk/uploads/score-sheet-gad-7-anxiety-and-phq-9-depression.pdf**7**](https://www.torbayandsouthdevon.nhs.uk/uploads/score-sheet-gad-7-anxiety-and-phq-9-depression.pdf7)

Haut du formulaire

**8/ You experienced a tragedy ….. months ago, with the decease of …………….**

**If you try to remember the moment of the death, could you describe me how you have been informed of the event?**

………………………………………………………………………………………………………………………………………………………………………………………………

………………………………………………………………

Did you receive a help proposition?

Yes No

00

00

If Yes, what kind of person proposed you some help (Policeman, Physician..)

…………………………………………………………………………………………………

and what kind of help?

…………………………………………………………………………………………………

And how (resources card, support …) ?

………………………………………………………………………………………………….

What kind of emotions did you feel during the 6-month after the decease?

1. ………………………
2. ………………………
3. ………………………
4. ………………………
5. ………………………

If we focus on the first emotion, could you tell me how long it lasted?

Duration of the 1st emotion ……………..

*Then follow with other emotions described*

Duration of the 2nd emotion……………..

Duration of the 3rd emotion……………..

Duration of the 4th emotion……………..

Duration of the 5th emotion……………..

**9/ Did you feel very anxious during this period?**

Yes No

00

00

If Yes, when were you the most anxious? ……………………………………

00

00

Did you need health care? Yes No

If Yes, which one? ………………………………………………………………………………

Or which if several ? ……………………………………………………………………………

Did it help you ? Yes No

00

00

If you did not use health care service, do you think now it would have been useful to ? Yes No

00

00

Why? …………………………………………………………………………………

00

00

Did you need medication ? Yes No

If Yes, which one? ……………………………………………………………………………

How long? ……………………………………………………………….

**10/ Did you feel, or do you still feel, rejected by the deceased ………. ?**

Yes No

00

00

**11/ Did you often ask « why did he/she make this? »?**

Yes No

00

00

Would you say that this question was even haunting you?

Yes No

00

00

Is it still haunting you?

Yes No

00

00

If not, how long was this question haunted you? ……………………….

**12/ Have you been involved in the suicide event? (receiving a call before his/her death; or in the search of the disappeared relative for example…)?**

Yes No

00

00

**13/Did you witness the suicide of your relative ?**

Yes No

00

00

Did you find your deceased relative?

Yes No

00

00

If Yes, do you have often some flashbacks of the event?

Yes No

00

00

Did these flashbacks prevent you from sleeping?

Yes No

00

00

Did you need a medication because of theses flashbacks?

Yes No

00

00

If Yes, which one? ………………………………………………………………………………

**14/ Would you have appreciate being reached by a professional to check your condition and to propose you some support?**

Yes No

00

00

Was it helpful?

Yes No

00

00

At what time after the suicide ?

……………………………………………..

**15/ What feelings did you have these last months?**

1. ………………………
2. ………………………
3. ………………………
4. ………………………

**16/ Were you able to talk about your sadness ?**

a/ with family?

Yes No

00

00

Was it helpful?

Yes No

00

00

If No, why?

………………………………………………………………………………………………………………………………………………………………………………………………

b/ With friends?

Yes No

00

00

Was it helpful?

Yes No

00

00

If No, why?

………………………………………………………………………………………………………………………………………………………………………………………………

c/ With neighbors?

Yes No

00

00

Was it helpful?

Yes No

00

00

If No, why?

………………………………………………………………………………………………………………………………………………………………………………………………

d/ With work mates?

Yes No

00

00

Was it helpful?

Yes No

00

00

If No, why?

………………………………………………………………………………………………………………………………………………………………………………………………

e/ A health worker?

Yes No

00

00

Was it helpful?

Yes No

00

00

If No, why?

………………………………………………………………………………………………………………………………………………………………………………………………

f/ In an association?

Yes No

00

00

Was it helpful?

Yes No

00

00

If No, why?

………………………………………………………………………………………………………………………………………………………………………………………………

**17/ If we focus on physical health, did you have an acute health problem over 3 weeks or chronic health problem these last 5 years?**

Yes No

00

00

This 2 last years in particular?

Yes No

00

00

If Yes, which? ……………………………………………………………………………

**18/ If we focus on mental health, did you have an acute health problem over 3 weeks or chronic health problem these last 5 years? For example, did you feel very anxious or depressed? Did you need medication for anxiety or depression ? Did you met a mental health professional ?**

Yes No

00

00

This 2 last years in particular?

Yes No

00

00

If Yes, which? ……………………………………………………………………………

**19/ Did you notice an increase in your tobacco consumption?**

Yes No

00

00

Alcohol consumption?

Yes No

00

00

**20/ In the beginning of the interview, we talked about brothers and sisters of …………….; (if applicable). Do you think that some of them would need to be reached to check his/her condition and to propose him/her some support?**

If Yes, in your opinion, did this person have been able to talk about his/her sadness?

Yes No

00

00

If Yes, what kind of help did he/she received? (*notice for each reported sources of help*)

……………………………………………………………………………………………………………………………………………………………………………………………………………………………………………………………………………………………………………………………………………………………………………………………………….....

If No, what kind of help would he/she need? (*notice for each reported kind of help*)

………………………………………………………………………………………………………………………………………………………………………………………………………………………………………………………………………………………………………………………………………………………………………………………………………

Do you think that we could reach this person together to propose help from our health services?

Yes No

00

00

If Yes, contact details of the person(s)

…………………………………………………………………………………………………………………………………………………………………………………………………
