# Supplementary material for: Being pro-active in meeting the needs of suicide-bereaved survivors: results from a systematic audit in Montréal
Source: BMC Public Health. 2020 Oct 10;20:1534. doi: 10.1186/s12889-020-09636-y (PMC7547412; doi:10.1186/s12889-020-09636-y)
Supplement: Supplementary file 2 — Additional file 2: Supplementary file 2. French Version of the semi-structured interview, GAD-7 and PHQ-9. [file 12889_2020_9636_MOESM2_ESM.docx]

## **Bordereau de recueil sur les endeuillés par suicide**

**AUDIT SYSTEMATIQUE DES CAS DE SUICIDE DU CIUSSS DE L’EST-DE-L’ILE DE MONTREAL**

**Questions à poser à l’endeuillé le concernant**

(Questions à poser après avoir parlé du défunt)

Age et genre de la personne décédée : ………………………………………………………..

Lien avec l’endeuillé : ………………………………………………………………………..

*« Nous allons maintenant parler de vous, de ce que vous avez traversé ces derniers mois, et ce afin de tenter de comprendre comment organiser les services afin qu’ils répondent au mieux aux besoins des personnes qui sont dans votre situation ».*

**Age : …………………………..**

**Genre :** Homme Femme

00

00

**1/ Travaillez-vous actuellement ?**

Oui Non

00

00

**2/ Quel est votre statut marital?**

En couple Séparé(e) Veuf(ve)

Célibataire

**3/ Avez-vous des enfants?**

Oui Non

00

00

Si oui quels âges ont-ils?

……………………….

……………………….

……………………….

……………………….

A quelle fréquence les voyez-vous ?

………………………………………………………………………………………………………………………………

**4/ (Questions autour du suicide)**

**Avez-vous d’autres proches que ………………… qui sont décédés par suicide?**

Oui Non

00

00

Si oui, qui (degré de parenté) et à quel âge?

……………………….

……………………….

Avez-vous des proches qui ont déjà fait une tentative de suicide?

Oui Non

00

00

Si oui, qui (degré de parenté) et à quel âge?

……………………….

……………………….

Avez-vous, vous-même, déjà fait une tentative de suicide ?

Oui Non

00

00

Si oui, à quel âge? (*Noter le nombre et chaque âge*)

……………………….

……………………….

**5/ Sur une échelle de 1 à 10, comment évalueriez-vous votre proximité relationnelle avec le défunt? (noter le chiffre donné s’il y en a un)**

00

00

**6/ PHQ-9**

**Au cours des deux dernières semaines, à quelle fréquence avez-vous été dérangé(e) par les problèmes suivants?**

(Encerclez votre réponse)

|  | Jamais | Plusieurs  jours | Plus de  la moitié  du temps | Presque  tous les  jours |
| --- | --- | --- | --- | --- |
| 1. Peu d’intérêt ou de plaisir à faire des choses | 0 | 1 | 2 | 3 |
| 2. Vous sentir triste, déprimé(e) ou désespéré(e) | 0 | 1 | 2 | 3 |
| 3. Difficultés à vous endormir ou à rester endormi(e), ou trop dormir | 0 | 1 | 2 | 3 |
| 4. Vous sentir fatigué(e) ou avoir peu d’énergie | 0 | 1 | 2 | 3 |
| 5. Peu d’appétit ou trop manger | 0 | 1 | 2 | 3 |
| 6. Mauvaise perception de vous‐même — ou vous pensez que vous êtes un perdant ou que vous n’avez pas satisfait vos propres attentes ou celles de votre famille | 0 | 1 | 2 | 3 |
| 7. Difficultés à vous concentrer sur des choses telles que lire le journal ou regarder la télévision | 0 | 1 | 2 | 3 |
| 8. Vous bougez ou parlez si lentement que les autres personnes ont pu le remarquer, ou au contraire, vous êtes si agité(e) que vousbougez beaucoup plus que d’habitude | 0 | 1 | 2 | 3 |
| 9. Vous avez pensé que vous seriez mieux mort(e) ou pensé à vous blesser d’une façon ou d’une autre | 0 | 1 | 2 | 3 |

Si la personne interrogée a répondu 1 ou plus à au moins un des problèmes nommés dans ce questionnaire, demander: « dans quelle mesure ce(s) problème(s) a‐t‐il (ont‐ils) rendu difficile votre travail, vos tâches à la maison ou votre capacité à bien vous entendre avec les autres? »

| Pas du tout  difficile | Plutôt  Difficile | Très  difficile | Extrêmement  difficile |
| --- | --- | --- | --- |
| 0 | 1 | 2 | 3 |

**7/ GAD-7**

**Au cours des deux dernières semaines, à quelle fréquence les problèmes suivants vous ont-ils inquiété ?** (Encercler les réponses) Haut du formulaire

|  | Jamais | Plusieurs  jours | Plus de  la moitié du temps | Presque tous les jours |
| --- | --- | --- | --- | --- |
| 1. Être nerveux, anxieux, les nerfs à vifs | 0 | 1 | 2 | 3 |
| 2. Incapable d’arrêter ou de contrôler son doute | 0 | 1 | 2 | 3 |
| 3. Douter trop de différentes choses | 0 | 1 | 2 | 3 |
| 4. Avoir de la difficulté à se relaxer | 0 | 1 | 2 | 3 |
| 5. Être si impatient qu’il est difficile de rester assis | 0 | 1 | 2 | 3 |
| 6. Devenez facilement énervé ou irrité | 0 | 1 | 2 | 3 |
| 7. Avoir peur que quelque chose d’horrible se produise | 0 | 1 | 2 | 3 |

Bas du formulaire

**8/ Vous avez vécu un drame il y a ….. mois avec la perte de ……..**

En vous replongeant au moment du décès de ….., pourriez-vous me décrire comment vous avez appris son décès?

Vous a-t-on alors proposé une aide?

Oui Non

00

00

Si oui, quelle personne vous a proposé une aide (policier, médecin…), quelle aide et de quelle manière (carte ressource, accompagnement…)?

Quelles émotions avez-vous ressenties dans les 6 mois suivant le décès?

1. ………………………
2. ………………………
3. ………………………
4. ………………………
5. ………………………

En reprenant la première émotion décrite, pourriez-vous me dire combien de temps elle a duré?

Durée de 1……………..

Poursuivre ensuite avec les autres états émotionnels rapportés par l’interviewé

Durée de 2……………..

Durée de 3……………..

Durée de 4……………..

Durée de 5……………..

**9/ Vous êtes-vous senti(e) très angoissé(e) à un moment (cocher la bonne réponse) ?**

Oui Non

00

00

Si oui, à quelle période surtout? ……………………………………

Avez-vous eu alors recours au service de soins? Oui Non

00

00

Si oui, lequel? ………………………………………………………………………………

Ou lesquels………………………………………………………………………………….

Est-ce que cela vous a aidé? Oui Non

00

00

Si vous n’avez pas eu recours à un service de soins, pensez-vous que cela vous aurait été bénéfique de consulter? Oui Non

00

00

Pourquoi ? …………………………………………………………………………………

Avez-vous eu recours à une médication ? Oui Non

00

00

Si oui, laquelle? ……………………………………………………………………………

Pendant combien de temps? ……………………………………………………………….

**10/ Avez-vous eu le sentiment, ou l’avez-vous encore, d’avoir été rejeté(e) par le défunt (ou la défunte)?**

Oui Non

00

00

**11/ Vous êtes-vous souvent posé la question du pourquoi concernant le geste réalisé?**

Oui Non

00

00

Diriez-vous que cette question était même obsédante, très présente ?

Oui Non

00

00

L’est-elle encore?

Oui Non

00

00

Sinon, pendant combien de temps vous a-t-elle envahi l’esprit? ……………………….

**12/ Avez-vous été impliquée dans les moments autour du suicide (appel du proche, recherche du proche…)?**

Oui Non

00

00

**13/Avez-vous assisté au suicide de votre proche ?**

Oui Non

00

00

Avez-vous découvert votre proche ?

Oui Non

00

00

Si oui, avez-vous des images qui vous reviennent souvent concernant cet évènement ?

Oui Non

00

00

Vous empêchent-elles de dormir?

Oui Non

00

00

Avez-vous eu besoin d’un traitement à cause de ces images?

Oui Non

00

00

Si oui, lequel? ………………………………………………………………………………

**14/ Auriez-vous apprécié d’être contacté(e) afin que l’on prenne de vos nouvelles et que l’on vous propose, éventuellement, une aide de type écoute?**

Oui Non

00

00

Auriez-vous considéré cela comme une aide?

Oui Non

00

00

A quel moment pensez-vous que cela aurait été le plus bénéfique pour vous?

……………………………………………..

**15/ Quels sentiments avez-vous ressentis ces derniers mois?**

1. ………………………
2. ………………………
3. ………………………
4. ………………………
5. ………………………

**16/ Avez-vous pu parler de votre peine**

a/ en famille?

Oui Non

00

00

Est-ce que cela vous a aidé?

Oui Non

00

00

Si non pourquoi?

………………………………………………………………………………………………………………………………………………………………………………………………

b/ avec des amis?

Oui Non

00

00

Est-ce que cela vous a aidé?

Oui Non

00

00

Si non pourquoi?

………………………………………………………………………………………………………………………………………………………………………………………………

c/ avec des voisins?

Oui Non

00

00

Est-ce que cela vous a aidé?

Oui Non

00

00

Si non pourquoi?

………………………………………………………………………………………………………………………………………………………………………………………………

d/ des collègues?

Oui Non

00

00

Est-ce que cela vous a aidé?

Oui Non

00

00

Si non pourquoi?

………………………………………………………………………………………………………………………………………………………………………………………………

e/ un soignant?

Oui Non

00

00

Est-ce que cela vous a aidé?

Oui Non

00

00

Si non pourquoi?

………………………………………………………………………………………………………………………………………………………………………………………………

f/ dans une association?

Oui Non

00

00

Est-ce que cela vous a aidé?

Oui Non

00

00

Si non pourquoi?

………………………………………………………………………………………………………………………………………………………………………………………………

**17/ Sur le plan de la santé physique, avez-vous présenté une maladie aigue de plus de 3 semaines ou chroniques ces 5 dernières années**?

Oui Non

00

00

Cette dernière année en particulier?

Oui Non

00

00

Si oui laquelle? ……………………………………………………………………………

**18/ Sur le plan de la santé psychique, avez-vous présenté une maladie aigue de plus de 3 semaines ou chronique ces 5 dernières années? Par exemple, avez-vous été très angoissé ou déprimé ? Avez-vous reçu des traitements pour l’anxiété ou la dépression? Avez-vous rencontré un professionnel de la santé mentale?**

Oui Non

00

00

Cette dernière année en particulier?

Oui Non

00

00

Si oui laquelle? ……………………………………………………………………………

**19/ Avez-vous noté une augmentation de votre consommation de tabac ces derniers mois?**

Oui Non

00

00

D’alcool?

Oui Non

00

00

**20/ Plus tôt, nous avons passé en revue les frères et sœurs du défunt *(si cela s’applique),* à votre connaissance, est-ce qu’il y a parmi eux quelqu’un qui aurait apprécié d’être contacté afin que l’on prenne de ses nouvelles et que l’on propose, éventuellement, une aide ?**

Si oui, à votre connaissance, cette personne a-t-elle pu parler de sa peine?

Oui Non

00

00

Si oui, quel type d’aide a-t-elle obtenue? Préciser pour chaque personne signalée

……………………………………………………………………………………………………………………………………………………………………………………………………………………………………………………………………………………………………………………………………………………………………………………………………….....

Si non, quel type d’aide aurait-elle besoin? Préciser pour chaque personne signalée

………………………………………………………………………………………………………………………………………………………………………………………………………………………………………………………………………………………………………………………………………………………………………………………………………

Croyez-vous que nous pourrions ensemble la contacter pour lui offrir de l’aide de nos services de santé?

Oui Non

00

00

Si oui, coordonnées de la personne concernée

…………………………………………………………………………………………………………………………………………………………………………………………………
